# Extreme ecological response of a seabird community to unprecedented sea ice cover

**DOI:** 10.1098/rsos.140456

**Published:** 2015-05-20

**Authors:** Christophe Barbraud, Karine Delord, Henri Weimerskirch

**Affiliations:** CEBC, UMR7372 CNRS, Villiers en Bois 79360, France

**Keywords:** Antarctic, breeding, sea ice, petrels, penguins, skuas

## Abstract

Climate change has been predicted to reduce Antarctic sea ice but, instead, sea ice surrounding Antarctica has expanded over the past 30 years, albeit with contrasted regional changes. Here we report a recent extreme event in sea ice conditions in East Antarctica and investigate its consequences on a seabird community. In early 2014, the Dumont d'Urville Sea experienced the highest magnitude sea ice cover (76.8%) event on record (1982–2013: range 11.3–65.3%; mean±95% confidence interval: 27.7% (23.1–32.2%)). Catastrophic effects were detected in the breeding output of all sympatric seabird species, with a total failure for two species. These results provide a new view crucial to predictive models of species abundance and distribution as to how extreme sea ice events might impact an entire community of top predators in polar marine ecosystems in a context of expanding sea ice in eastern Antarctica.

## 2. Introduction

Recent analyses of trends in observational climate record witnessed that, in contrast to Arctic sea ice, Antarctic sea ice has undergone a paradoxical increase over the last 30 years [1,2]. Increasing trends in sea ice extent, concentration and season duration were observed in most parts of Antarctica (except in the lower-latitude Western Antarctic Peninsula where the opposite situation occurs). These trends have been linked to changes in atmospheric dynamics [3–5] influenced by the Antarctic ozone hole [6], and by freshening of the Antarctic Ocean owing to melting of the ice sheet and ice shelves in response to climate warming [7].

Given the utmost importance of sea ice in the functioning of the Antarctic Ocean ecosystems [8–10], the Antarctic marine biota may be affected by the increase in sea ice, but it is not known which way the ecosystems may respond [11]. Indeed, there is a noticeable absence of empirical studies addressing whether an increase in sea ice affects Antarctic marine ecosystems positively or negatively. Also lacking are studies addressing whether climate extremes can potentially affect polar marine ecosystems, and which key variables might be involved. As climate is changing rapidly in polar ecosystems [11] and since polar marine ecosystems are influenced by climate and weather fluctuations at comparable or greater rates that terrestrial ecosystems [12], it is very likely that the occurrence and impact of extreme events has been severely under-reported in these ecosystems.

Here, using data derived from a 50 year long-term observational study of top predator responses to climate change in the Southern Ocean, we report an unprecedented high sea ice concentration event and investigate its ecological consequences on an entire seabird community.

## 3. Material and methods

### 3.1. Sea ice

The monthly sea ice concentration data for the Dumont d'Urville Sea area (66° S–67° S, 139° E–142° E) were obtained from a suite of satellite-based passive microwave sensors provided by the National Snow and Ice Data Center and available at the International Research Institute for Climate and Society. This area corresponds to the main foraging area of the seabird species breeding at the Pointe Géologie archipelago during summer [13–15]. Sea ice concentration is the fraction of area covered by sea ice. Sea ice data were obtained from http://iridl.ldeo.columbia.edu/SOURCES/.NOAA/.NCEP/.EMC/.CMB/.GLOBAL/.Reyn_SmithOIv2/.monthly/.sea_ice [16] for January 1982 through to April 2014. Monthly data were averaged for the Dumont d'Urville Sea and were separated into two groups: summer (December–March) and other seasons (April–November) to examine the extreme event of the 2013–2014 austral summer. To identify the presence and timing of a significant change in sea ice concentration in these grouped time series, we applied a Davies test (*k*=30) using the ‘segmented’ package in R [17,18]. This tests for a value at which a significant difference in slope can be identified through the use of Wald statistics corrected for repeated testing across *k* evenly spaced potential break points [17].

### 3.2. Breeding success

Seabird breeding success has been monitored at the Pointe Géologie archipelago (66°40^′^ S, 140°01^′^ E) situated on the edge of the Antarctic continent and adjacent to the Dumont d'Urville Sea since the early 1950s. Breeding success was defined as the proportion of eggs laid producing a fledging for species that lay a single egg and as the number of fledged chicks per breeding pair for two egg laying species. For southern fulmars, snow petrels and Wilson storm petrels nest sites were monitored and the numbers of eggs laid, chicks hatched and chicks fledged were recorded (southern fulmar: approx. 60 nest sites [19], i.e. entire colony; snow petrels: approx. 70 to approx. 300 nest sites [20]; Wilson storm petrel: approx. 70 nest sites). For south polar skuas, the total numbers of breeding pairs, chicks hatched and chicks fledged were counted on the entire archipelago during visits on the territories every two weeks. For Adélie penguins, the numbers of breeding pairs, chicks hatched and chicks fledged were counted on the main island of the archipelago, which host approximately 80% of the total breeding population in the archipelago [21]. For emperor penguins, the number of chicks fledged was counted at the end of the breeding season. During the entire breeding season, numbers of chicks and eggs found dead nearby the colony were counted on a daily basis and used retrospectively to estimate the number of breeding pairs and chicks hatched [22]. We investigated the relationships between seabird breeding success and sea ice concentration in the Dumont d'Urville Sea during summer using non-parametric smoothing regression techniques [23]. Generalised Additive Models (GAM) were specified with a Gaussian family, using a penalized cubic regression spline, and the optimal amount of smoothing was estimated using cross-validation. The adjusted *R*-squared for the model was defined as the proportion of variance explained, where original variance and residual variance were both estimated using unbiased estimators. This quantity could be negative if the fitted model was worse than a one-parameter constant model [23].

## 4. Results

**Figure 1.**
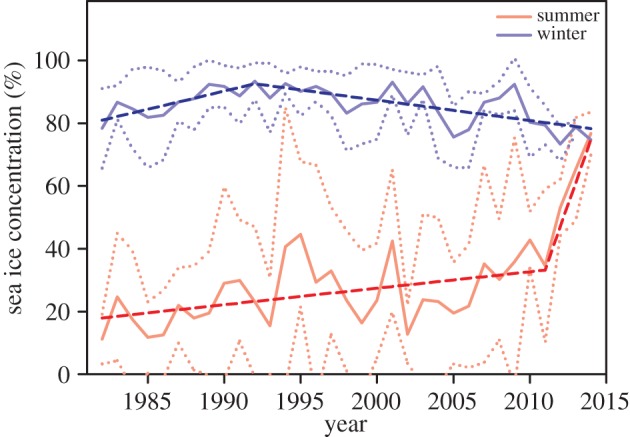
Monthly sea ice concentration in the Dumont d'Urville Sea colour coded by season (summer: December–March; winter: April–November). The rate of increase in monthly sea ice concentration increased significantly in summer beginning in 2011 (red dashed line) and the sea ice concentration in winter started to decrease in 1992 (blue dashed line). Dotted lines indicate ±s.d. calculated on the variation between months.

Time series of summer (December–March) sea ice concentration exhibited a significant change towards more sea ice since 1982, with more rapid sea ice increase around the year 2011 (95% confidence interval (CI): 2010, 2012; Davies tests, *k*=30, *p*<0.001), together with a significant but slight sea ice loss during winter since the year 1992 (95% CI: 1989, 1995; Davies test, *k*=30, *p*<0.001). Following this summer trend in sea ice increase, the Dumont d'Urville Sea experienced in January and February 2014 unprecedented sea ice concentrations 473% above normal (figure 1). For the period 1982–2013, the average sea ice concentration in January and February in the Dumont d'Urville Sea was 13.9±14.3% (min: 0.2%; max: 51.7%) but it reached 79.7% in early 2014. The sea ice concentration anomaly persisted for two months along the coastline with extensive areas of fast ice remaining throughout the austral summer in most parts of the coastline.

**Figure 2.**
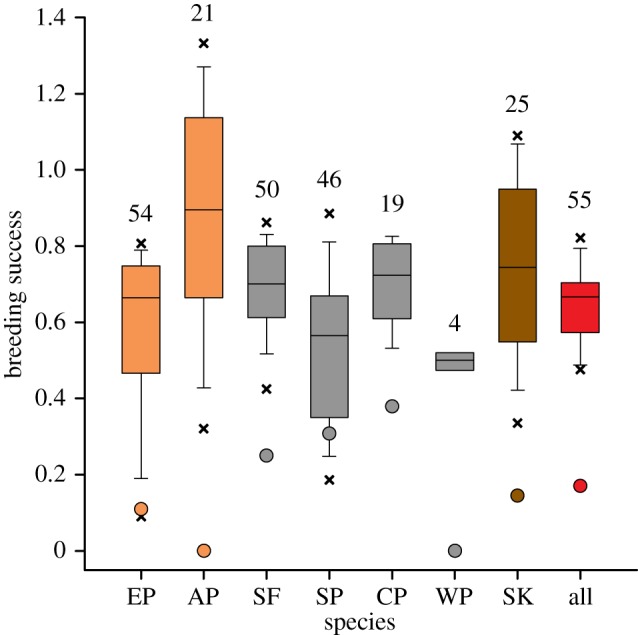
Box plots of breeding success of emperor penguins (EP), Adélie penguins (AP), southern fulmars (SF), snow petrels (SP), cape petrels (CP), Wilson storm petrels (WP), south polar skuas (SK) and all species combined (all). The band inside the box is the median, the bottom and top of the box are the 25% and 75% quartiles, respectively, the lower and upper whiskers are the 10% and 90% quartiles, respectively, the lower and upper crosses are the 5% and 95% quartiles, respectively. Dots indicate breeding success data for the 2013–2014 breeding season. Colours indicate penguins (orange), petrels (black), skuas (brown) and all (red). Numbers indicate the number of years breeding success was recorded. Breeding success was defined as the proportion of eggs laid producing a fledging for species that lay a single egg and as the number of fledged chicks per breeding pair for two egg laying species (AP and SK).

The entire seabird community exhibited a common response to the 2014 extreme event (figure 2). All the seven species' breeding success dropped to very low values, well below the observed range of breeding successes observed since the beginning of the long-term monitoring program 50 years ago. For five species, the breeding success of the 2013–2014 breeding season was the lowest ever recorded, and two of them totally failed to even fledge a single chick (Adélie penguin *Pygoscelis adeliae* and Wilson's storm petrel *Oceanites oceanicus*). This resulted by far in the lowest average breeding success for the community (figure 2). For two species most of the failures occurred during the incubation stage (snow petrel *Pagodroma nivea*: 95% of failures; southern fulmar *Fulmarus glacialoides*: 100%), for one species most of the failures occurred during the chick stage (emperor penguin *Aptenodytes forsteri*: 67.5%), whereas for two other species failures occurred during both the egg and the chick stage (Adélie penguin: 56.8%; south polar skua *Catharacta maccormicki*: 54.2%).

**Figure 3.**
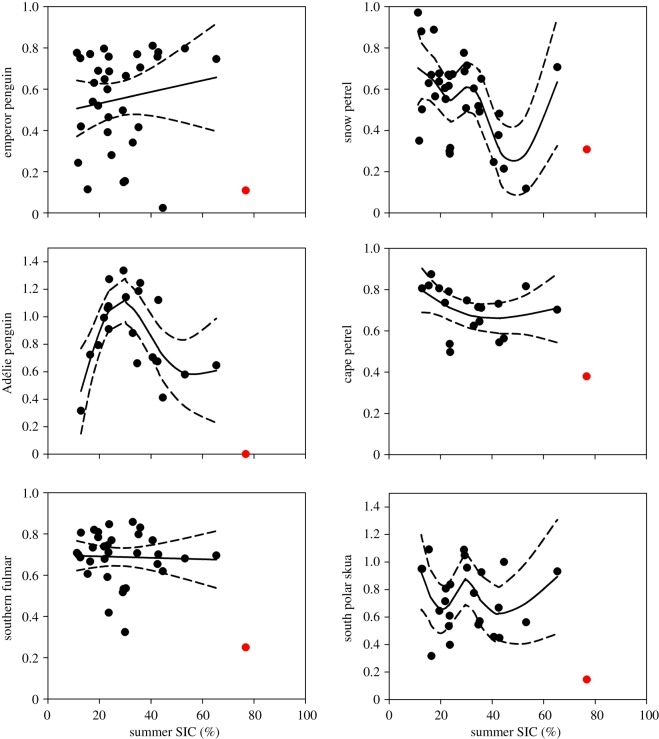
Fitted GAM results showing the relationship between the seabird breeding success data and the summer sea ice concentration (SIC) in the Dumont d'Urville Sea for the period 1982–2013. Dotted lines indicate 95% CIs. Red dots indicate the observed breeding success in 2014. Breeding success was defined as the proportion of eggs laid producing a fledging for species that lay a single egg and as the number of fledged chicks per breeding pair for two egg laying species (Adélie penguin and south polar skua.).

Penguins had a lower success (0.055±0.055) than flying seabirds (0.216±0.066, *z*=1.88, *p*=0.06). For the period 1982–2014, thus including the extreme sea ice concentration year of 2014, breeding success was negatively related to sea ice concentration in summer for most summer breeding species, but not for the only species that breeds in winter, the emperor penguin (table 1). However, for the period 1982–2013, only Adélie penguin and snow petrel breeding success was negatively related to sea ice concentration in summer (table 1 and figure 3).

**Table 1.** GAM results for the breeding success if six seabird species from the Pointe Géologie archipelago as a function of summer sea ice concentration in the Dumont d'Urville Sea. (EP, emperor penguin; AP, Adélie penguin; SF, southern fulmar; SP, snow petrel; CP, cape petrel; SK, south polar skua; All, average breeding success for all species combined. Numbers in parentheses indicate pairs of degrees of freedom.)

	without 2014			with 2014		
species	*F*-test	*p*-value	adjusted *R*^2^	*F*-test	*p*-value	adjusted *R*^2^
EP	0.684 (1, 1)	0.415	−0.010	0.122 (1, 1)	0.729	−0.028
AP	4.759 (3.48, 4.20)	0.009	0.509	6.919 (3.95, 4.72)	0.001	0.615
SF	0.038 (1, 1)	0.847	−0.032	2.854 (1.88, 2.31)	0.066	0.154
SP	3.282 (4.56, 5.33)	0.017	0.337	4.795 (1.21, 1.38)	0.026	0.172
CP	1.426 (1.93, 2.39)	0.268	0.155	3.697 (5.65, 6.47)	0.022	0.540
SK	1.124 (4.76, 5.63)	0.384	0.134	1.665 (5.29, 6.15)	0.183	0.228

## 5. Discussion

This study constitutes, to our knowledge, one of the few empirical instances of an impact of an extreme climatic event on an entire community of top predators in a polar marine ecosystem [24]. It concurs with the recent evidence that extreme climatic events can synchronize population parameters at the community level as in terrestrial ecosystems [25].

Several lines of evidence strongly suggest that the extremely low breeding success of the seabird community was driven by extreme high sea ice concentrations. First, there is an association between most species' breeding success and sea ice concentration in Terre Adélie and in other sites in eastern Antarctica [26]. Second, the community of seabirds forage within the Dumont d'Urville Sea during the breeding period in loose pack ice areas and in open waters in the vicinity of the ice edge [13–15] where prey are most abundant [27]. December–February constitutes the period when adult foraging is most intense, having to incubate their egg and feed their chicks. An extreme sea ice cover during this period may increase foraging costs, forcing parents to cover longer distances than usual to reach foraging areas. This would increase incubation fasts and body mass loss of adults and reduce meal frequency for the chicks, leading to increases in nest parental desertion and chick mortality, as reported for some Arctic seabird species [28]. Such a process would be particularly acute for non-flying birds such as penguins for which long range movement capacities are more limited. Indeed, the breeding success for penguins was lower than that of flying birds. This was particularly dramatic for Adélie penguins which usually fledge between 15 000 and 30 000 chicks each year but not even a single chick in 2014. As a consequence, south polar skuas, for which Adélie penguin eggs and chicks constitute an important food resource during the breeding season, experienced their lowest breeding success ever recorded, illustrating a cascading effect on apex predators. Skuas were not able to feed their chick following the massive dying of Adélie penguin chicks. For emperor penguins, the unusually high mortality of the chicks was linked to the large distances on fast ice travelled by the adults between the colony and the foraging grounds from October to January (approx. 80–90 km) in line with earlier findings [29]. Among flying species those feeding in open water and loose pack ice [30] (southern fulmar, cape petrel *Daption capense* and Wilson storm petrel) were particularly affected, and the least affected species was the pagophilic snow petrel. Finally, extreme sea ice concentration may have increased protection from predators for prey species using sea ice as a refuge and which are commonly consumed by seabirds (such as the Antarctic silverfish *Pleuragramma antarctica* and krill, *Euphausia* spp.), thereby enhancing difficulties for predators to access food resources.

Importantly, models fitted to breeding success and summer sea ice concentration data, excluding the last year of unprecedented sea ice concentration (2014), predicted completely different responses to the 2014 high sea ice concentration compared with the observed responses. For example, although the model for the Adélie penguin breeding success had a good fit for the period 1982–2013 (R adjusted=0.509, table 1), the predicted breeding success for 2014 was 0.67, whereas the observed breeding success was 0. Therefore, the response observed on breeding success was not only extreme but also unexpected based on models relating breeding success and sea ice concentration. This also clearly indicates that, when making projections, extrapolation of the explanatory environmental variables to values well outside the observed parameter range is problematic and dangerous [31,32].

These results are of primary interest for future studies investigating the ecological impacts of climate change and extreme events. Antarctic sea ice projections from general circulation models suggest a sea ice loss by the end of the twenty-first century [34,35]. Recent modelling suggest that this sea ice loss is likely to have negative impacts on Antarctic top predators and ecosystems [34,35]. However, by the mid-twenty-first century, melting of the Antarctic ice sheet and ice shelves could be changing the vertical ocean stratification around Antarctica and encourage sea ice growth [7], as supported by the observed trends [12]. Sea ice growth may increase the frequency and intensity of extreme sea ice events such as the one documented here. Our study suggests that a transitional sea ice growth by the mid-twenty-first century would greatly amplify the catastrophic projected impact of the sea ice loss by the end of the century on Antarctic marine predators and ecosystems, with diminished populations having to face a substantial retreat in sea ice. However, the long-term consequences of such a transient phase with sea ice growth will depend on the length of the transient phases. For example, there could be a phase with favourable sea ice concentrations after the phase with high sea ice concentrations, which may favour population growth of emperor penguins. Alternatively, during the transient phase with high sea ice concentrations, populations may move to more favourable sites situated in areas with optimal sea ice concentrations. In addition, it is at present unclear whether sea ice conditions recorded in early 2014 in the Dumont d'Urville Sea correspond to an extreme event with conditions returning to a normal state within the coming years or whether this constitutes a more permanent shift to new sea ice conditions. In the latter case, this would have an even higher considerable impact on seabirds and the marine ecosystem in the near term.

## Data Accessibility

Supporting data and R scripts are deposited in Dryad, http://dx.doi.org/10.5061/dryad.3c2v9.
